# H3.3K122A results in a neomorphic phenotype in mouse embryonic stem cells

**DOI:** 10.21203/rs.3.rs-4824795/v1

**Published:** 2024-08-27

**Authors:** Benjamin Patty, Cailin Jordan, Santana Lardo, Kris Troy, Sarah Hainer

**Affiliations:** University of Pittsburgh; University of Pittsburgh; University of Pittsburgh; University of Pittsburgh; University of Pittsburgh

## Abstract

The histone variant H3.3 acts in coordination with histone posttranslational modifications and other chromatin features to facilitate appropriate transcription. Canonical histone H3 and histone variant H3.3 are post-translationally modified with the genomic distribution of these marks denoting different features and with more recent evidence suggesting that these modifications may influence transcription. While the majority of posttranslational modifications occur on histone tails, there are defined modifications within the globular domain, such as acetylation of H3K122/H3.3K122. To understand the function of the residue H3.3K122 in transcriptional regulation, we attempted to generate H3.3K122A mouse embryonic stem (mES) cells but were unsuccessful. Through multi-omic profiling of mutant cell lines harboring two or three of four H3.3 targeted alleles, we have uncovered that H3.3K122A is neomorphic and results in lethality. This is surprising as prior studies demonstrate H3.3-null mES cells are viable and pluripotent, albeit with reduced differentiation capacity. Together, these studies have uncovered a novel dependence of a globular domain residue of H3.3 for viability and broadened our understanding of how histone variants contribute to transcription regulation and pluripotency in mES cells.

## INTRODUCTION

Individual canonical histone proteins within a nucleosome can be substituted with histone variants (reviewed in^1^), which play important roles in chromatin dynamics during DNA-templated activities^1,2^. Variant histone proteins for each of the four canonical histone proteins have been identified, with among the most well studied being histone variant H3.3, which replaces canonical histone H3. In mammals, there are two genes that encode H3.3: *H3f3a* and *H3f3b*^2^. *H3f3a* and *H3f3b* encode identical polypeptide sequence, but exhibit allele-specific gene structures, nucleic acid sequences, expression patterns, and have different requirements during development^3–6^. The amino acid sequence of variant H3.3 is very similar to that of canonical H3.1 and H3.2, with only five differing amino acids^1^. H3.3 has a context-specific relationship with transcription, and is found at regions of active transcription such as enhancers and regions of heterochromatin including telomeres and other repeat regions^7–10^. The context specific role of H3.3 is mediated by distinct histone chaperone systems and post-translational modifications: the HIRA complex incorporates H3.3 into euchromatin where it receives transcription associated modifications such as acetylation, while chromatin remodeler ATRX and histone chaperone DAXX incorporate H3.3 into heterochromatic regions where it receives methylation marks associated with repression^8,9,11–13^. At these locations, H3.3 regulates the turnover of nucleosomes to promote proper transcription factor (TF) binding and/or the maintenance of histone modifications to preserve the local chromatin state^14–17^. In murine embryonic stem cells (mES cells), H3.3 is dispensable for viability and regulation of pluripotency, but is required for differentiation and transitions between cell states^10,13,18,19^.

Active enhancers are characterized by distinct chromatin features, including flanking nucleosomes marked by H3K4me1 and H3K27ac^20,21^. However, beyond these well-established histone modifications, there are additional modifications that are less well characterized. K122 is a residue found at the nucleosome dyad in both H3 and H3.3 and can be acetylated in mammals and fission yeast (H3K122ac)^22^. *In vitro*, H3K122ac reduces histone/DNA interactions by destabilizing nucleosomes^23^. Though budding yeast lack H3K122ac, H3K122A, H3K122R, and H3K122Q mutations reduce nucleosome occupancy over highly transcribed genes and result in reduced telomeric, ribosomal, and HML silencing^24,25^. Finally, the Bickmore lab found that in mES cells and MCF-7 human cells H3K122ac marks a subset of enhancers, sometimes without H3K27ac^26^. Together, these data suggest an important role for H3K122 in eukaryotic cells, with the implication that acetylation may stimulate transcription. However, to date, there has been no study dissecting how H3.3 and K122ac may coordinate to regulate transcription in eukaryotes.

To examine the role of H3.3K122 in mES cells, we attempted to generate mES cells containing *H3f3a* and *H3f3b* K122A homozygous mutations. In addition to altering residue charge and size, a mutation that replaces the lysine residue with an alanine (H3.3 K122A) would prevent the deposition of the H3.3 K122ac as alanine residues are not readily acetylated. Despite many attempts to generate mES cells where all four H3.3 alleles contain the K122A mutation, we were unable to recover these cell lines, suggesting lethality. We succeeded in generating cell lines containing either two or three of the four H3.3 alleles with the K122A mutation. We were unable to deplete the expression of the remaining WT H3.3 within these cell lines, supporting that having H3.3K122A as the sole H3.3 protein is lethal. Despite this, to investigate the impact of H3.3K122A on mES cells, we characterized cell lines containing two or three of four alleles with the K122A mutation. We found that with increasing numbers of H3.3K122 alleles targeted, the phenotypic, transcriptomic, and epigenomic defects are compounded. Together, these studies suggest that K122ac is critical to proper H3.3 function in mES cells.

## RESULTS

### H3.3K122A may be lethal in mES cells

**Figure 1:**
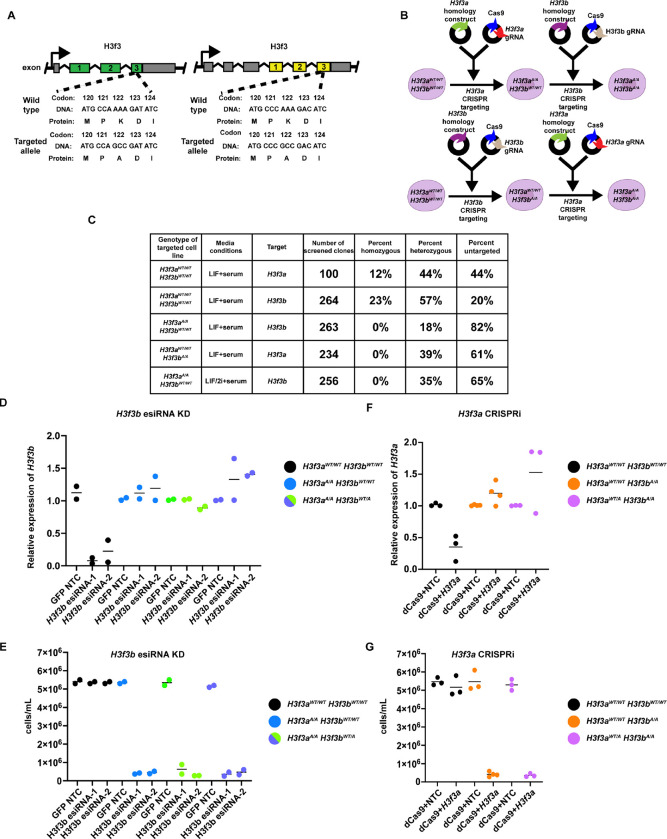
H3.3K122A mutation may be lethal in ES cells. A. Schematic of *H3f3a* (left) and *H3f3b* (right) loci, with WT and targeted DNA sequences denoted. B. Schematic of two-step targeting method to generate H3.3 K122A cell lines. C. Table summarizing the mES cell clones screened to generate single targeted cell lines (top two rows) and dual targeted cell lines (bottom four rows). “Media conditions” indicates the media formula used during targeting. A full description of clones screened can be found in the Methods and in Supplemental Table 1. D. RT-qPCR results of esiRNA knockdowns targeting *H3f3b* or GFP non-template control (NTC) in wildtype cell lines (control for KD efficiency), *H3f3a*^A/A^ *H3f3b*^WT/WT^ cell lines (parental), and *H3f3a*^A/A^ *H3f3b*^WT/A^ cell lines. Each point is an independent biological replicate. The line represents the average of each biological replicates. E. As in D, for RT-qPCR results of CRISPRi targeting of *H3f3a* or non-template control (NTC) in wildtype cell lines (control for efficiency), *H3f3a*^WT/WT^*H3f3b*^A/A^ cell lines (parental), and *H3f3a*^WT/A^*H3f3b*^A/A^ cell line. F. Number of viable cells for esiRNA knockdowns targeting *H3f3b* or GFP non-template control (NTC) in wildtype cell lines (control for KD efficiency), *H3f3a*^A/A^ *H3f3b*^WT/WT^ cell lines (parental), and *H3f3a*^A/A^ *H3f3b*^WT/A^ cell lines. Each point is an independent biological replicate. The line represents the average of biological replicates. G. As in F, for CRISPRi targeting of *H3f3a* or non-template control (NTC) in wildtype cell lines (control for efficiency), *H3f3a*^WT/WT^ *H3f3b*^A/A^ cell lines (parental), and *H3f3a*^WT/A^ *H3f3b*^A/A^ cell lines.

While *H3f3a* and *H3f3b* encode the same polypeptide, the gene structures and coding sequences of *H3f3a* and *H3f3b* are distinct (Fig. 1A). Therefore, we used a two-step targeting strategy to generate mutations in *H3f3a* and *H3f3b*. First, we independently targeted either *H3f3a* or *H3f3b* using CRISPR/Cas9-directed homologous recombination to replace the endogenous sequence with a single codon change to alter lysine 122 to alanine (K122A; Fig. 1B). We were able to generate homozygous single targeted lines (*H3f3a*^A/A^*H3f3b*^WT/WT^ or *H3f3a*^WT/WT^*H3f3b*^A/A^ cell lines) with reasonable frequency (Fig. 1C and Supplemental Table 1). Sequence-confirmed *H3f3a*^A/A^*H3f3b*^WT/WT^ and *H3f3a*^WT/WT^*H3f3b*^A/A^ cell lines (referred to as single targeted) were then independently targeted for the opposite H3.3 encoding gene, with the goal of generating cell lines containing all four alleles with the K122A mutation (Fig. 1B and Supplemental Table 1). Despite multiple attempts at generating *H3f3a*^A/A^ *H3f3b*^A/A^ cell lines in both LIF/serum and 2i + LIF/serum media, we were unable to generate these clones (Fig. 1C and Supplemental Table 1). However, we did obtain dual targeted (either *H3f3a*^WT/A^*H3f3b*^A/A^ or *H3f3a*^A/A^*H3f3b*^WT/A^) cell lines (Fig. 1C and Supplemental Table 1).

Given these results, we hypothesized that *H3f3a*^A/A^*H3f3b*^A/A^ may be lethal in mES cells. To test this, we attempted to deplete remaining wildtype H3.3 using esiRNAs or CRISPRi in single targeted (*H3f3a*^WT/WT^*H3f3b*^A/A^ or *H3f3a*^A/A^*H3f3b*^WT/WT^) and dual targeted (*H3f3a*^WT/A^*H3f3b*^A/A^ or *H3f3a*^A/A^*H3f3b*^WT/A^) cell lines. We designed and tested two sets of esiRNAs targeting *H3f3b* (we were unable to design unique esiRNAs targeting *H3f3a* that resulted in depletion) and transfected these into wildtype, *H3f3a*^A/A^*H3f3b*^WT/WT^, or *H3f3a*^A/A^*H3f3b*^WT/A^ cell lines using esiRNAs targeting GFP as an unexpressed non-template control (NTC). After 48 hours, we observed 85–95% depletion of *H3f3b* in wildtype cells using either set of *H3f3b*-targeting esiRNAs, yet no depletion of *H3f3b* in cell lines harboring *H3f3a*^A/A^ and *H3f3b*^WT/WT^ or *H3f3b*^WT/A^ (Fig. 1D). We believe this is due to lethality upon successful *H3f3b* depletion in these cell lines, where we observed ~ 10-fold reduction in viable cells in *H3f3a*^A/A^ and *H3f3b*^WT/WT^ or *H3f3b*^WT/A^ relative to *H3f3a*^WT/WT^*H3f3b*^WT/WT^ and *H3f3a*^WT/A^*H3f3b*^WT/WT^ cell lines (Fig. 1E). We then designed and generated sgRNAs targeting *H3f3a* and performed CRISPRi in wildtype, *H3f3a*^WT/WT^*H3f3b*^A/A^, and *H3f3a*^WT/A^*H3f3b*^A/A^ cell lines (Fig. 1F). We observed ~ 55% depletion of *H3f3a* only in wildtype cells, but no depletion of *H3f3a* in cell lines harboring *H3f3b*^A/A^ (Fig. 1F). Similar to esiRNA targeting, CRISPRi targeting of *H3f3a* in *H3f3a*^WT/WT^*H3f3b*^A/A^ and *H3f3a*^WT/A^*H3f3b*^A/A^ cells resulted in an approximate 10-fold reduction in abundance of viable cells relative to controls (Fig. 1G). Together with our inability to generate *H3f3a*^A/A^*H3f3b*^A/A^ clones, these results suggest that the H3.3K122A mutation at all four H3.3 alleles may be lethal in mES cells. These results are surprising as it has been previously shown that H3.3 KO mES cells and mES cells depleted of H3.3 are viable^10,18^. Despite these findings, to further understand the role of H3.3K122 in mES cells, we characterized cell lines containing either two or three of the H3.3 alleles targeted for H3.3K122A.

### H3.3K122A results in reduced pluripotency and slow growth in mES cells

**Figure 2:**
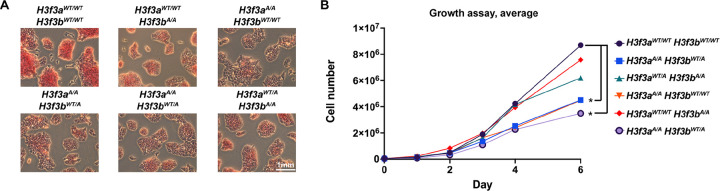
H3.3K122A cell lines have reduced AP staining and growth rate relative to wildtype cells. A. Alkaline phosphatase staining of wildtype and H3.3K122A cell lines. Shown are representative images from one of three biological replicates. B. Growth curve for WT and H3.3K122A cell lines with live cell numbers quantified at 1, 2, 3, 4, and 6 days post plating. Graph represents the average of 3 biological replicates.

To understand how the H3.3 K122A mutation impacts mES cell biology, we assessed whether mES cell pluripotency was disrupted using alkaline phosphatase (AP) staining (Fig. 2A, Supplemental Fig. 1A). AP is a membrane-bound protein highly expressed in mES cells, and is commonly used as a phenotypic proxy for pluripotency^27^. All mutant cell lines displayed reduced AP staining relative to wildtype, with *H3f3a*^A/A^ or *H3f3a*^WT/A^ containing cell lines showing slightly less staining relative to *H3f3a*^WT/WT^ containing cell lines (Fig. 2A, Supplemental Fig. 1A). We next performed a 6-day growth assay with wildtype and mutant cell lines (Fig. 2B, Supplemental Fig. 1B-D). Though all mutant cell lines had a minor reduction in growth by day 6 relative to wildtype, *H3f3a*^A/A^ containing cell lines had a more consistent and severe growth defect relative to *H3f3a*^WT/WT^ or *H3f3a*^WT/A^ containing cell lines (Fig. 2B, Supplemental Fig. 1B-D). Interestingly, these data support a previous study arguing that *H3f3a* is evolutionarily specialized for transcriptional programs associated with cell proliferation^5^. Together, these observations may suggest allele-specific requirements in the functions of H3.3K122 related to cell proliferation and regulation of pluripotency, with *H3f3a* having a more prominent role in mES cells.

### H3.3K122A mES cells have reduced levels of H3.3

**Figure 3:**
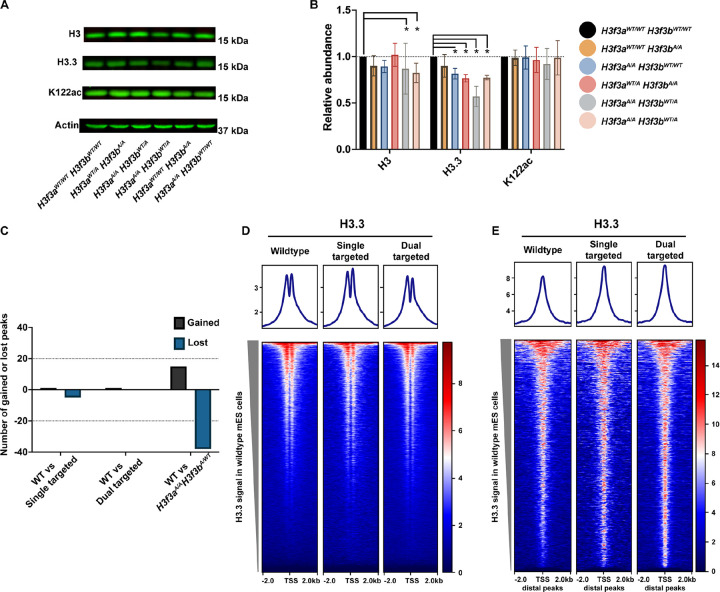
H3.3K122A cell lines have reduced H3.3 protein levels relative to wildtype cells but not reduced H3.3 chromatin occupancy. A. Western blotting for total H3, H3.3, and K122ac in wildtype and H3.3K122A cell lines. Actin is used as a loading control. B. Quantification of three biological replicates for each Western blot in A. Each dot represents a single biological replicate. *=P value ≤0.05, two sided T-test. C. Differentially enriched H3.3 CUT&RUN peaks (|LFC|≥0.5 and FDR≤0.05) in the single targeted and dual targeted cell lines relative to wildtype. Black indicates number of upregulated peaks, and blue indicates number of downregulated peaks. D. Metaplots and heatmaps visualizing average H3.3 enrichment over TSSs in wildtype, single targeted, and dual targeted cell lines. N=22,598 loci. E. As in F, for gene distal H3.3 peaks. N=3,347 peaks.

We next examined the protein levels of H3, H3.3, and K122ac (both H3K122ac and H3.3K122ac) in each cell line and observed little change in H3 and K122ac abundance (Fig. 3A-B). Interestingly, all *H3f3a*-targeted cell lines (*H3f3a*^WT/A^ or *H3f3a*^A/A^) exhibited a modest reduction in H3.3 abundance (~ 60–70% of WT levels) while the *H3f3a*^WT/WT^*H3f3b*^A/A^ lines had little or no reduction (Fig. 3A-B). We performed total and nascent RT-qPCR and found that all cell lines exhibited similar levels of *H3f3a*, and found the *H3f3b*^A/A^ cell lines exhibited reduced expression and transcription of *H3f3b* (Supplemental Fig. 1E-F). Therefore, while all targeted lines examined have reduced abundance of H3.3 protein, only *H3f3b*^A/A^ cell lines have reduced gene expression.

### H3.3K122A cells display no major perturbations in H3.3 occupancy

Given our finding that H3.3 protein levels are reduced in the mutant cell lines, we examined whether H3.3 occupancy is altered. To that end, we performed Cleavage Under Target and Release Using Nuclease (CUT&RUN) for H3.3 in wildtype mES cells and each single/dual targeted cell line, with high reproducibility between replicates and across cell lines (Supplemental Fig. 1G). We defined 5,447 H3.3 consensus peaks across the datasets (excluding repetitive regions) yet found less than 60 H3.3 peaks with differential enrichment in all comparisons to wildtype (Fig. 3C). This implies that, while the targeted cell lines have reduced protein abundance of total H3.3 relative to wildtype, these cell lines have maintained similar levels of H3.3 within chromatin as wildtype cells. Visualization of H3.3 enrichment at transcription start sites (TSSs) and H3.3 gene distal peaks showed similar enrichment in wildtype, single targeted, and dual targeted cell lines, recapitulating this finding (Fig. 3D-E).

### Severity of transcription perturbations upon H3.3K122A mutation increases with number of alleles targeted

**Figure 4:**
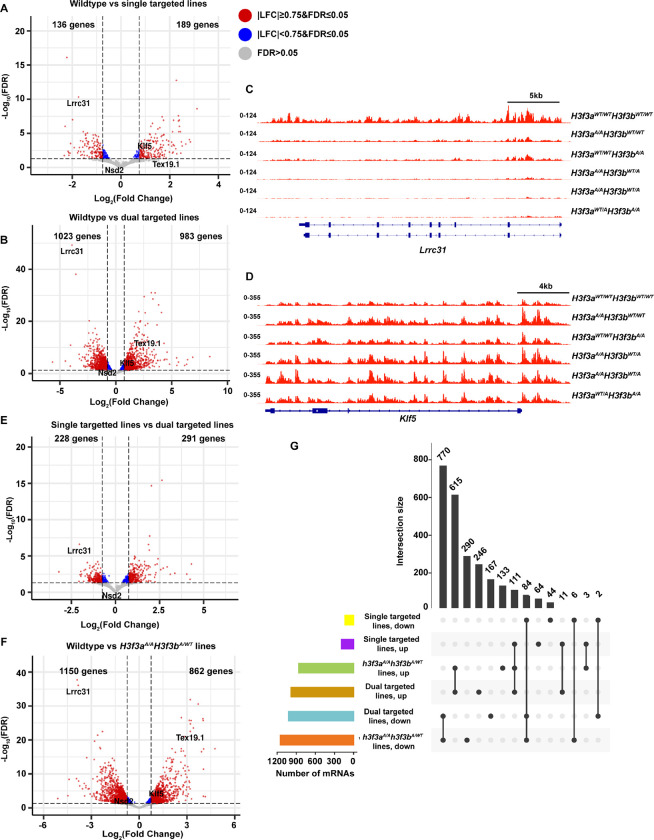
H3.3K122A results in transcriptomic changes in ES cells. A. Volcano plot of differentially transcribed genes in single targeted H3.3K122A cells (*H3f3a*^A/A^*H3f3b*^WT/WT^ and *H3f3a*^WT/WT^*H3f3b*^A/A^) relative to wildtype. Red points are genes |LFC|≥0.75 and FDR≤0.05, blue points are genes |LFC|<0.75 and FDR≤0.05, and grey points are not significantly changed. B. As in A, for dual targeted lines (*H3f3a*^A/A^*H3f3b*^A/WT^ and *H3f3a*^A/WT^*H3f3b*^A/A^) relative to wildtype. C. Browser track of nascent RNA-seq (TT-seq) from H3.3K122A cell lines and wildtype cells over the *Lrrc31* locus. D. As in E, for the *Klf5* locus. E. As in A, for dual targeted relative to single targeted cell lines. F. As in A, for *H3f3a*^A/A^*H3f3b*^A/WT^ lines relative to wildtype. G. Upset plot showing the number of differentially transcribed genes (log_2_(FC)≥0.75 and FDR≤0.05) shared between set of groups (upper bar graph) and the overall number of differentially transcribed genes in each group (lower left bar graph).

To understand how H3.3K122A impacts the mES cell transcriptome, we performed the nascent RNA sequencing method Transient Transcriptome sequencing (TT-seq^28^) in wildtype and H3.3K122A targeted cell lines with high reproducibility (Supplemental Fig. 2A). When comparing the single targeted (*H3f3a*^A/A^*H3f3b*^WT/WT^ and *H3f3a*^WT/WT^*H3f3b*^A/A^) cell lines to wildtype, we observed transcriptional changes in hundreds of genes, with 136 downregulated and 189 upregulated (Fig. 4A). However, when comparing dual targeted (*H3f3a*^A/A^*H3f3b*^WT/A^ and *H3f3a*^WT/A^*H3f3b*^A/A^) cell lines to wildtype, we detected thousands of genes with altered transcription (1023 downregulated and 983 upregulated; Fig. 4B). We selected a panel of genes upregulated (*Klf5* and *Tex19.1*) and downregulated (*Nsd2* and *Lrcc31*) and validated these trends with nascent and total RT-qPCR in all targeted cell lines (Supplemental Fig. 2B-E). Visual comparison of TT-seq data across the *Lrrc31* and *Klf5* locus in wildtype and mutant cell lines further validated these trends and indicated a relationship between the severity of transcriptional perturbation and the number of targeted H3.3 alleles in the genetic background (Fig. 4C-D). We compared transcriptional changes between the single and dual targeted cell lines, observing an intermediate number of genes with perturbed transcription as expected (Fig. 4E). Given that we performed TT-seq on two clones with identical genotypes (*H3f3a*^A/A^ *H3f3b*^WT/A^), we also compared the *H3f3a*^A/A^ *H3f3b*^WT/A^ dual targeted lines relative to wildtype and found 1150 downregulated and 862 upregulated genes (Fig. 4F).

We next compared how these differentially expressed genes (DEGs) compared across single targeted (*H3f3a*^A/A^*H3f3b*^WT/WT^ and *H3f3a*^WT/WT^*H3f3b*^A/A^) and dual targeted cell lines, where we examined dual targeted as either *H3f3a*^A/A^*H3f3b*^WT/A^ only (two lines) or *H3f3a*^A/A^*H3f3b*^WT/A^ and *H3f3a*^WT/A^*H3f3b*^A/A^ (three lines; Fig. 4G). Moving forward, we will distinguish these genotypes as dual targeted when referring to results combined from *H3f3a*^A/A^*H3f3b*^WT/A^ and *H3f3a*^WT/A^*H3f3b*^A/A^ and as *H3f3a*^A/A^*H3f3b*^WT/A^ when referring to results from these lines only.

We next compared differentially expressed genes (DEGs) in single targeted (*H3f3a*^A/A^*H3f3b*^WT/WT^, *H3f3a*^WT/WT^*H3f3b*^A/A^) and dual targeted cell lines (*H3f3a*^A/A^*H3f3b*^WT/A^ alone or with *H3f3a*^WT/A^*H3f3b*^A/A^; Fig. 4G). We’ll refer to results from *H3f3a*^A/A^*H3f3b*^WT/A^ and *H3f3a*^WT/A^*H3f3b*^A/A^ as “dual targeted” and results from *H3f3a*^A/A^*H3f3b*^WT/A^ alone as *H3f3a*^A/A^*H3f3b*^WT/A^.

When comparing the dual targeted vs *H3f3a*^A/A^*H3f3b*^WT/A^ DEGs we found that the dual targeted vs wildtype had 246 upregulated and 167 downregulated unique genes relative to *H3f3a*^A/A^*H3f3b*^WT/A^ (Fig. 4E). These unique DEGs therefore likely stem from the *H3f3a*^WT/A^*H3f3b*^A/A^ line, thus allowing an opportunity to examine allele-specific effects. To that end, we performed gene ontology (GO) on the overlapping and unique gene sets from the *H3f3a*^A/A^*H3f3b*^WT/A^ vs wildtype and dual targeted vs wildtype comparisons (Supplemental Fig. 2F). Genes upregulated and downregulated in both comparisons were enriched in terms related to metabolism and lineage commitment toward vasculogenesis. Genes uniquely downregulated in the *H3f3a*^A/A^*H3f3b*^WT/A^ lines were enriched for processes related to neuronal lineage commitment, while unique upregulated genes were associated with epithelial lineage commitment. Together, these studies further support allele-specific requirements for *H3f3a* and *H3f3b*.

#### Dual targeted cell lines exhibit strong K27ac and subtle K122ac enrichment changes relative to single targeted or wildtype mES cells

**Figure 5:**
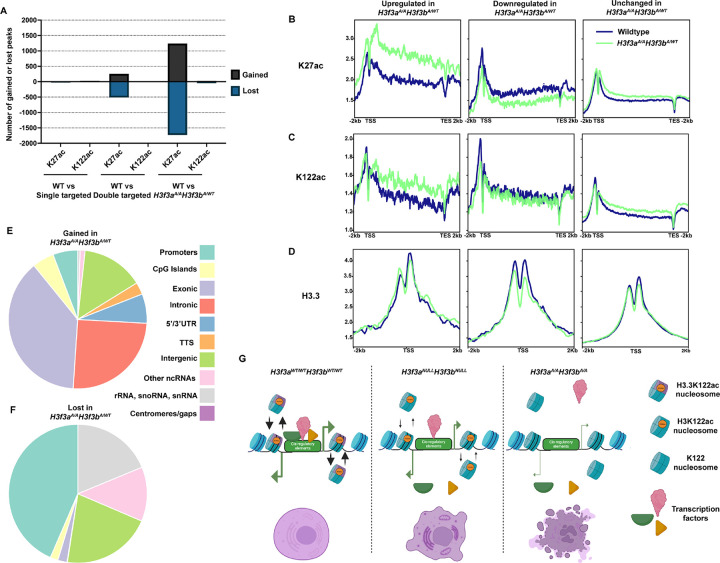
*H3f3a*^A/A^ *H3f3b*^A/WT^ cell lines exhibit altered patterns of K27ac enrichment relative to wildtype mES cells. A. Bar graph showing the number of differentially enriched K27ac or K122ac CUT&RUN peaks (|LFC| ≥0.5 and FDR≤0.05) for the indicated cell line comparisons. B. Metaplots of K27ac enrichment over genes upregulated, downregulated, or unchanged in *H3f3a*^A/A^*H3f3b*^A/WT^ cells and wildtype mES cells. C. As in B, for K122ac enrichment. D. As in B, for H3.3 enrichment. E. Pie chart of the HOMER annotation of gained K27ac peaks in *H3f3a*^A/A^*H3f3b*^A/WT^ mES cells relative to wildtype. Each color corresponds to a genomic feature in the legend. F. As in E, for lost K27ac peaks in *H3f3a*^A/A^*H3f3b*^A/WT^ mES cells relative to wildtype. G. Model for how H3.3K122A may result in lethality in mES cells.

To evaluate how the H3.3K122A mutation influences the epigenome of mES cells, we profiled the enrichment of K27ac (antibody recognizes both H3K27ac and H3.3K27ac) and K122ac (antibody recognizes both H3K122ac and H3.3K122ac) using CUT&RUN in wildtype and each single targeted and dual targeted line with high reproducibility (Supplemental Fig. 3A-B). For K122ac, we found fewer than 100 peaks with differential enrichment in the single targeted or dual targeted lines relative to wildtype (Fig. 5A). In contrast to K122ac, we observed few differentially enriched K27ac peaks in the single targeted cell lines, hundreds in dual targeted cell lines relative to wildtype, and thousands when comparing *H3f3a*^A/A^*H3f3b*^WT/A^ lines to wildtype (Fig. 5A). Notably, these data follow a similar trend to the transcriptomic changes (Fig. 4), with increasing severity as more targeted H3.3K122 alleles are present.

We next examined K122ac and K27ac enrichment over our consensus K27ac peaks and K122ac peaks called from published ChIP-seq datasets^26^ in wildtype, single targeted, and dual targeted cell lines. We found that while wildtype and single targeted cell lines had comparable levels of K27ac enrichment, the dual targeted cell lines exhibited higher levels of K27ac over consensus peaks (Supplemental Fig. 3C). However, both single and dual targeted cell lines exhibited lower levels of K122ac over K122ac ChIP-seq peaks relative to wildtype (Supplemental Fig. 3D). As we predict the targeted cell lines to have reduced abundance of H3.3K122ac in targeted cell lines, but the K122ac antibody does not distinguish H3.3K122ac from H3K122ac, these findings suggest that H3.3K122ac may represent a substantial fraction of the global H3K122ac found in mES cells.

#### H3.3K122A does not disrupt H3 acetylation or H3.3 enrichment over protein coding genes in targeted cell lines

We next examined how H3K27ac enrichment changes in the *H3f3a*^A/A^*H3f3b*^WT/A^ lines might relate to the transcription defects observed. Specifically, we compared K27ac, K122ac, and H3.3 enrichment in the *H3f3a*^A/A^*H3f3b*^WT/A^ lines and wildtype mES cells at the promoters of genes with significantly changed transcription. Prior studies demonstrate that H3/H3.3 acetylation at promoters and over protein coding gene bodies generally correlates with transcription levels and changes in gene expression^29–32^. Reflecting their changed expression, the promoters and gene bodies of upregulated genes exhibited increased K27ac and a modest increase in K122ac in *H3f3a*^A/A^*H3f3b*^WT/A^ lines relative to wildtype, while downregulated genes have similar decreased levels of both marks over promoters and gene bodies (Fig. 5B-C). In *H3f3a*^A/A^*H3f3b*^WT/A^ lines, upregulated genes exhibited similar changes in H3.3 enrichment at their promoters relative to wildtype while downregulated genes exhibited a modest reduction in H3.3 enrichment consistent with decreased expression (Fig. 5D). Visual comparison of K27ac, K122ac, H3.3, and nascent transcription in the *H3f3a*^A/A^*H3f3b*^WT/A^ and wildtype mES cells over the Acad9 and Adam19 loci validate these trends (Supplemental Fig. 4A-B). Therefore, while the H3.3K122A mutation alters transcription in mES cells, it does not appear to interfere with H3 acetylation or H3.3 enrichment at promoters or over protein coding gene bodies.

### H3.3K122A leads to epigenetic changes over putative enhancers regions involved in regulation of cell fate

To examine the TSS distal effects of the H3.3K122A mutation, we determined the genomic locations of the gained and lost K27ac peaks in the *H3f3a*^A/A^*H3f3b*^WT/A^ lines. Interestingly, while 44% of lost K27ac peaks were associated with promoters, only approximately 6% of gained K27ac peaks were promoter-associated (Fig. 5E,F). Rather, 38%, 25%, and 14% of gained K27ac peaks were annotated as exonic, intronic, and intergenic, respectively, showing that the majority of regions with increased K27ac in the *H3f3a*^A/A^*H3f3b*^WT/A^ lines are TSS distal (Fig. 5E,F). Given this distinction, we compared the enrichment of K27ac, K122ac, and H3.3 over gained, lost, and unchanged TSS distal K27ac peaks in wildtype and *H3f3a*^A/A^*H3f3b*^WT/A^ lines (Supplemental Fig. 4C-E). Gained and lost peaks show enrichment of K27ac, K122ac, and H3.3 in either wildtype or *H3f3a*^A/A^*H3f3b*^WT/A^ mES cells, with lost K27ac exhibiting initially higher levels of K27ac, K122ac, H3.3, and chromatin accessibility (from ATAC-seq) relative to gained or unchanged peaks in wildtype mES cells (Supplemental Fig. 4C-F). Gained and lost TSS distal K27ac peaks displayed the expected changes in K27ac and K122ac enrichment in *H3f3a*^A/A^*H3f3b*^WT/A^ mES cells (Supplemental Fig. 4B-D), suggesting that these are putative enhancers with altered activity in the *H3f3a*^A/A^*H3f3b*^WT/A^ mES cells. Interestingly, gained TSS distal K27ac peaks exhibited an increase in H3.3 levels while lost peaks exhibited a reduction but still maintained H3.3 enrichment (at levels similar to gained or unchanged peaks; Supplemental Fig. 4E). As with the promoter profiles, these changes in enrichment likely reflect changes in transcriptional activity, suggesting that the H3.3K122A mutation also does not interfere with H3 acetylation or H3.3 enrichment at putative enhancers in mES cells. In addition, we found that lost TSS distal K27ac peaks in the *H3f3a*^A/A^*H3f3b*^WT/A^ were strongly enriched for motifs associated with regulation of pluripotency (including SOX family motifs and the OCT4/SOX2/NANOG/TCF motif) that did not have similar representation at gained promoter distal K27ac peaks (Supplemental Fig. 4G). Together, these analyses suggest that the H3.3K122A mutation influences the activity of a subset of putative enhancers in mES cells, and this may contribute to the mRNA expression changes and pluripotency defects observed in the *H3f3a*^A/A^*H3f3b*^WT/A^ mES cells.

## Discussion

The role of H3.3 in mES cells has been extensively characterized, showing that H3.3 and its associated post-translational modifications are important for regulation of endogenous retroviruses, heterochromatin, global H3 acetylation, and facilitating differentiation^7,9,10,13,16,18,19,33,34^. However, these studies also demonstrate that H3.3 is dispensable for proper transcription and regulation of pluripotency in mES cells. Specifically, depletion or deletion of H3.3 is not associated with proliferative defects, reduction in pluripotency, or strong transcriptional phenotypes^10,13,18,33^, arguing that the effects we observe are not due to reduced levels of H3.3 and are rather attributed to the H3.3K122A mutations.

Our findings highlight an importance for H3.3K122 in viability and maintenance of mES cells, but the exact mechanism remains elusive. *H3f3a*^A/A^*H3f3b*^WT/A^ mES cells still acquire K27ac and K122ac in a transcription-dependent manner, arguing that this residue does not regulate H3K27 acetylation dynamics as has been shown for H3.3S31P^13^. Our findings indicate that differences in activity of putative enhancers between *H3f3a*^A/A^*H3f3b*^WT/A^ and wildtype mES cells may play a role in the observed transcriptional defects of the targeted cell lines. Incorporation of H3.3 at regions of active transcription promotes nucleosome turnover, which has been shown to be important for TF binding, histone modification maintenance, and proper regulation of transcription^1,2^. Likewise, H3K122ac promotes nucleosome eviction *In vitro* and is necessary for rapid transcription activation in *S. pombe*^22^. Therefore, H3.3K122 may play an important role in H3.3 turnover dynamics and the H3.3K122A mutation may disrupt this process. We envision that, whereas mES cells lacking H3.3 have less nucleosome turnover but still enough for viability through use of H3K122, H3.3K122A can be integrated into nucleosomes but disrupts nucleosome turnover to point that the result is lethality (Fig. 5G). The correlation between the number of targeted H3.3 alleles and severity of phenotypes observed in mutant cell lines supports this model, but further studies are required.

## METHODS

### Cell culture

ES-E14TG2a (E14) mES cells from male *Mus musculus* origin (RRID:CVCL9108361^35^) were cultured as previously described^36^ at 37°C and 5% CO2 in DMEM base medium (Sigma Aldrich) supplemented with 10% FBS(Sigma Aldrich), 1X nonessential amino acids (Corning), 2mM L-glutamine (Corning), β-mercaptoethanol (Acros Organics) and LIF on 10cm plates precoated with 0.2% gelatin. For LIF + 2i media, LIF media was further supplemented with 3 μM CHIR99021 GSK inhibitor (p212121), and 1 μM PD0325091 MEK inhibitor (p212121). Cells were passaged every ~ 48 h using trypsin and split at a ratio of ~ 1:8 with fresh medium. Routine anti-mycoplasma TC hood cleaning was conducted (LookOut DNA Erase spray, Sigma Aldrich) and cell lines were screened to confirm no mycoplasma presence. While generating clones, cells were grown in media with the addition of 1X penicillin/streptomycin (Corning) to prevent bacterial contamination.

### Guide RNA cloning

Homology constructs for *H3f3a* and *H3f3b* (Supplemental Table 3) were designed to mutated residue Lysine 122 to Alanine and mutated the PAM sequence. Oligos generated for the *H3f3a* or *H3f3b* gRNA were phosphorylated and annealed, then ligated to the px330-puro plasmid backbone using a simultaneous Fast Digest *Bbs1* (ThermoFisher) digestion and Quick Ligase ligation (NEB). Plasmids were purified using the GenElute HP Plasmid Maxiprep Kit (Sigma Aldrich) following the manufacturer’s instructions and confirmed using Sanger sequencing (Genewiz).

Homology constructs were synthesized using IDT gBlocks Gene Fragments (IDT). Lyophilized construct was resuspended in 5 μL of nuclease-free water, and 1 μL was used to clone the construct into the TOPO vector using the Zero Blunt TOPO PCR Cloning Kit for Sequencing (ThermoFisher) according to the manufacturer’s directions. The resulting plasmids were purified using the GenElute HP Plasmid Maxiprep Kit (Sigma Aldrich) and confirmed using Sanger sequencing (Genewiz).

### H3.3K122A cell line generation

The targeting strategy used to generate cell lines where all four H3.3 alleles were mutated from lysine 122 to alanine is detailed in Fig. 1A. To generate cell lines, 2×10^5^ cells were plated in a single well in a 6 well dish 24 hours prior to transfection. 1 hour prior to transfection, medium was replaced with fresh, antibiotic-free medium. 3 μg of pX330-puro Cas9/guide RNA vector and 3 μg of recombination template vector were mixed with 100 μL of pre-warmed OptiMEM (Gibco). 24 μL of FuGENE HD (Promega) was added and the mixture was incubated at room temperature for 10 minutes. The mixture was added in drops evenly around the well of the dish and then gently swirled to ensure even mixing. 14–16 hours post-transfection, cells were split using 0.5% trypsin (Gibco) and plated at varying densities on gelatinized 10cm plates in antibiotic-free media. 48 hours post-transfection, the medium was replaced with medium containing 2 μg/mL puromycin. 72 hours post-transfection, the medium was replaced with antibiotic-free media. Individual clones were picked 8 days post-transfection. Two days after picking clones, media was replaced with fresh penicillin/streptomycin media. Five days after picking clones, cells were split to 3 identical plates to allow for gDNA extraction and genetic screening and parallel storage for post-screening use. Targeting was performed in LIF and LIF + 2i containing media.

### Clone screening

To extract gDNA, cells were washed once in 1xDPBS, lysed with ES cell lysis buffer (10 mM Tris pH 7.5, 10 mM EDTA, 10 mM NaCl, and 0.5% sarkosyl in water) including 1 mg/mL proteinase K, and incubated overnight at 55°C. The following day, plates were centrifuged for 2 minutes at 4°C and 1000rpm. DNA was precipitated using ice cold 100% ethanol with 75 mM NaCl and incubated for about 30 minutes until the solution cleared before centrifuging for 5 minutes at 4°C and 3000rpm. The supernatant was removed and the DNA was washed twice with 70% ethanol, then dried for an hour and resuspended in 50 μL 1X TE. PCR was performed on the extracted DNA in 96-well plates using primers that amplified the targeted *H3f3a* or *H3f3b* region (see Supplemental Table 2).

### esiRNA generation and knockdown

Endoribonuclease-digested short interfering RNAs (esiRNAs) were generated as previously described^36^. An ideal target DNA sequence was identified within *H3f3b* and screened for unique nucleotide sequence via DEQOR. siRNAs were *In vitro* transcribed from cDNA collected from wildtype mES cells using T7 RNA polymerase. To generate esiRNAs, IVT products were digested with ShortCut RNase III (NEB) and purified using a PureLink RNA Mini Kit (Invitrogen). Transient transfections to attempt to deplete *H3.3b* were performed on wildtype cells (control for transfection efficiency) or *H3f3a* K122A clones on 6 wells using 5 μL of Lipofectamine 3000 (ThermoFisher) and 900 ng of esiRNAs for 48 hours. Cells were harvested and counted with trypan blue on a BioRad Cell Counter. Following RNA isolation, depletion was quantified using RT-qPCR.

### CRISPRi

Guide RNA for *H3f3a* were cloned into dCas9-KRAB-MeCP2 plasmid (Addgene 110821), as previously described^37^. dCas9-KRAB-MeCP2 was a gift from Alejandro Chavez & George Church (Addgene plasmid # 110821; http://n2t.net/addgene:110821; RRID:Addgene 110821). Plasmids were transfected into wildtype mES cells (control for transfection efficiency and CRISPRi activity) or *H3f3b* K122A clones on 6 wells using 5 μL of Lipofectamine 3000 (ThermoFisher), 5 μL of p3000 reagent, and 5 μg of plasmid for 48 hours. Cells were harvested and counted with trypan blue on a BioRad Cell Counter. Following RNA isolation, transcript abundance was quantified using RT-qPCR.

### Protein extraction and western blotting

For each protein, the following primary/secondary antibodies and dilutions were used: H3 (abcam ab1791 GR300978–2, 1:1000), H3.3 (Millipore 09–838 3987735, 1:1000), K122ac (Invitrogen PA5–112508 YJ4090633A, 1:1000), and ACTIN (Sigma A1978 037M4782V, 1:5000), IRDye 800CW Goat anti-rabbit IgG Secondary (LI-COR 926–32211, 1:10000), and IRDye 800CW Goat anti-mouse IgG Secondary (LI-COR 926–32210, 1:10000).

For H3, cells were trypsinized and collected in a 15 mL conical tube, then spun down at 600 rcf for 5 min at 4°C, and protein was extracted using 100 μL RIPA buffer (150 mM NaCl, 1% IPEGAL CA-630, 0.5% sodium deoxycholate, 0.1% sodium dodecyl sulfate, and 25 mM Tris–Cl, pH 7.4) with protease inhibitors (ThermoFisher). For H3.3 and K122ac, cells were trypsinized and collected in a 15 mL conical tube, then spun down at 600 rcf for 5 min at 4°C. Cell pellets were washed twice with cold 1XPBS and protein was extracted using 200 μL Triton Extraction Buffer (PBS containing 0.5% Triton X 100 (v/v), 2 mM phenylmethylsulfonyl fluoride (PMSF), 0.02% (w/v) NaN3). Concentration of all protein extracts were determined using the Pierce BCA protein assay kit (ThermoFisher), and 10–30 μg protein per sample were loaded onto 15% Tris-acrylamide gels with Precision Plus ladder (Biorad). Gels were run for 40V for 1 hour, 120V for 2.5 hours, and then transferred at 20V overnight to a nitrocellulose membrane (Biotrace). Total protein was determined using REVERT 700 total protein stain (LI-COR) and blocked using Intercept blocking buffer (LI-COR). Primary antibody incubations were conducted overnight at 4°C, and secondary antibody incubations were conducted for 2 hours at room temperature. All imaging was done using a LI-COR Odyssey DLx Imager according to the manufacturers specifications. Protein quantification was conducted as previously described using ImageJ^38^. For total protein quantification, identical areas for each lane were selected and the mean pixel intensity was measured and subtracted from the mean pixel background intensity. For target protein quantification, identical size areas immediately surrounding the target band were selected and mean pixel intensity was measured and subtracted from mean background pixel intensity. Target protein quantification was then made relative to total protein quantification, and then made relative to wildtype. Three biological replicates were conducted per target for each cell line.

### Cell growth assay

Cells were plated in 6 wells at a density of 50,000 cells per well. At days 1, 2, 3, 4, and 6 post-plating, cells were trypsinized using 0.5 mL of trypsin and stopped with 1.5 mL of ES cell medium. After achieving single cell suspension, cells were counted on a TC20 cell counter (BioRad) using trypan blue stain to distinguish live cells. Three biological replicates were conducted for each cell line.

### Alkaline phosphatase staining

Cells were washed twice in 1X Dulbecco’s Phosphate-Buffered Saline (DPBS, Gibco) and crosslinked for five minutes at room temperature using 1% formaldehyde (Fisher) in DPBS. Crosslinking was quenched using 500 mM glycine and cells were washed using 1X DPBS twice. Cells were stained using Vector Red Alkaline Phosphatase Staining Kit (Vector Labs) per manufacturer’s instructions in a 200 mM Tris-HCl buffer, pH 8.4. 2 mL working solution was added to each 6 well and incubated in the dark for 30 minutes before being washed with 1XPBS and imaged. Three biological replicates were conducted for each cell line.

### 4sU-labelling and RNA isolation

4sU-labelled RNA was generated and isolated from cells using the TT-seq approach as previously described^28,39^. The media was aspirated from cell plates and replaced with 10 mL of 500 nM 4-thiouridine (4sU, Carbosynth T4509) containing ES cell media and the plates incubated at 37°C with 5% CO_2_ for 5 min. After 5 min, the 4sU-containing media was aspirated and the cells were washed with 1xPBS, and 2 mL TRIzol (Invitrogen) was added directly to the plate. Cell lysate were collected, and RNA was extracted according to ThermoFisher’s recommendations. RNA concentration was determined by Qubit with the Qubit RNA broad range quantification kit (ThermoFisher). 100 μg of total RNA was diluted to a concentration of 240 ng/μL at a volume of 416.67 μL in 1XTE and then fragmented with a Diagenode Bioruptor Pico on high power for one 30 second cycle. The fragmented RNA was then combined with 283.33 μL 1XTE, 100 μL 10X Biotinylation buffer (100 mM Tris pH 7.4 and 10 mM EDTA), and 200 μL of 1 mg/mL biotin-HPDP (ThermoFisher) in dimethylformamide (DMF; freshly prepared). Samples were vortexed, then incubated in a thermomixer at 37°C shaking at 1000 RPM in the dark for 2 hours. Samples were then chloroform extracted, isopropanol/salt precipitated, and resuspended in 22 μL of nuclease-free water. 60 uL of Streptavidin C1 beads (Invitrogen) were prepared for RNA separation by washes with 1 mL of 1 M NaOH and 50 mM NaCl and resuspended in 60 μL of TT-seq Binding buffer (10 nM Tris pH 7.4, 300 mM NaCl, 0.1% Triton). Then, 60 μL of prepared streptavidin C1 beads were added to each sample and rotated at room temperature for 20 min. Following incubation, the samples were magnetized for 1 min and the supernatant (containing the unlabeled RNA) was placed in a separate 1.5 mL tube and put on ice. The unlabeled RNA from supernatant was Phenol:Chroloform:Isopropanol(PCI)/chloroform extracted, isopropanol/salt precipitated, and resuspended in 100 μL of nuclease-free water. The bead-bound labeled nascent RNA was washed twice with 500 μL of High Salt buffer (50 mM Tris pH 7.4, 2M NaCl, 0.5% Triton), twice with 500 μL of TT-seq Binding buffer, and once with 500 μL of Low Salt buffer (5 mM Tris pH 7.4, 0.1% Triton), rotating for 1 min at room temperature, re-magnetizing and resuspending the beads during each wash. The nascent RNA was recovered from the beads through two rounds of 100 μL of freshly prepared 100 mM DTT and incubating in a thermomixer at 65°C and 1000 RPM shaking for 5 min. Eluted nascent RNA was recovered with a PCI extraction and an isopropanol/salt/glycogen precipitation. RNA pellets were resuspended in 25 μL of nuclease-free water. The total RNA and nascent RNA from each sample were used for RT-qPCR and TT-seq libraries as described below.

### Transient Transcriptome sequencing (TT-seq)

For TT-seq, three replicates for wildtype mES cells, and one replicate for each mutant cell line were performed. 4sU labelling for TT-seq was performed as described in 4sU labelling section. RNA pellets were resuspended in 1X TE buffer, at approximately 100 μL per 100 μg of RNA. Strand-specific nascent RNA library building was performed as previously described^39^ using the NEBNext Ultra II Directional Library kit with the following changes: The rRNA depleted RNA was fragmented at 94°C for fifteen minutes following the manufacturer’s instructions for intact RNA, the fragmentation, first strand cDNA synthesis, and second strand cDNA synthesis were performed at double the reaction volume in the manufacturer’s instructions, the adaptors were diluted 1:5 in Adaptor Dilution Buffer, the primers were diluted 1:5 in nuclease-free water, and 7 cycles of PCR were used to amplify the libraries. Finished libraries were quantified by Quibit with the dsDNA High Sensitivity kit and run on a Fragment Analyzer to confirm high quality of each library prior to sequencing. Libraries were sequenced paired-end on an Illumina NextSeq500 to ~ 40 million mapped reads.

### TT-seq analysis

Raw paired end fastq files were analyzed with fastQC^40^ using default parameters to ensure high data quality and adapter sequences were trimmed using cutadapt^41^. Reads were aligned with the mm10 Gencode annotation (GRCm38.p6, vM23) using STAR^42^ with the following parameters: outFilterMismatchNoverReadLmax 0.02, outFilterMultimapNmax 1. samtools^43^ was used to filter reads and generate indexed bams with the following parameters: -q 7 -f 2. Feature counts were generated using featurecounts^44^ options -B -t “exon” -g “gene_name” -F GTF -p -s 2 with the mm10 (GRCm38.p6, vM23) genome annotation (GTF format). Differential gene expression analysis was conducted using DEseq2^45^ with cutoff as |log2(FC)|≥0.75 and FDR ≤ 0.05. Gene Ontology analysis on gene sets of interest was performed the R package “clusterProfiler”^46^ using all Gencode features as background. Upset plots were generated using the R package “upsetR” (https://github.com/hms-dbmi/UpSetR), and volcano plots were generated using R package “EnhancedVolcano” (https://bioconductor.org/packages/release/bioc/html/EnhancedVolcano.html).

### Reverse transcription and quantitative PCR (RT-qPCR)

cDNA was generated from 1 μg of total RNA or 100 ng of 4sU labelled RNA with random hexamers (Promega) with homemade reverse transcriptase (RT). Generated cDNA was used as a template in qPCR reactions using 2X SYBR FAST mix (KAPA) and 5 μM target specific primers (see Supplemental Table 2) on a Lightcycler 96 (Roche). Target transcript abundance in samples was determined using the ΔΔCT normalization method relative to wildtype samples, using *Gapdh* transcript abundance for internal normalization as previously described^47^.

### CUT&RUN

The following antibodies and dilutions were used for CUT&RUN: H3.3 (Active Motif 91191, lot # 01022005; 1:50), K122ac (Invitrogen PA5–105108, lot # WJ3411065A; 1:50), and K27ac (abcam ab4729, lot # GR3416784–1; 1:100).

CUT&RUN was performed as described^39,48,49^ using recombinant ProteinA-MNase (pA/G-MN) or Protein A/Protein G-MNase (pA/G-MN), as specified. For each target, two replicates for wildtype and one replicates for each mutant cell line were performed. Briefly, 100,000 nuclei were isolated from cell populations using a hypotonic buffer (20 mM HEPES-KOH, pH 7.9, 10 mM KCl, 0.5mM spermidine, 0.1% Triton X-100, 20% glycerol, freshly added protease inhibitors) and flash frozen. Nuclei were thawed and bound to lectin-coated concanavalin A magnetic beads (50 μL bead slurry per 100,000 nuclei; Polysciences). Immobilized nuclei were pre-blocked with blocking buffer (20 mM HEPES, pH 7.5, 150 mM NaCl, 0.5mM spermidine, 0.1% BSA, 2 mM EDTA, fresh protease inhibitors) and washed in wash buffer (20 mM HEPES, pH 7.5, 150 mM NaCl, 0.5mM spermidine, 0.1% BSA, fresh protease inhibitors). Nuclei were incubated in wash buffer containing primary antibody (H3.3, K122ac, K27ac) overnight at 4°C with rotation. Nuclei were incubated in wash buffer containing primary antibody for 1 hour at room temperature with rotation. Next, samples were washed twice with wash buffer and incubated in wash buffer containing recombinant pA-MN (for K122ac, K27ac) or pA/G-MN (for H3.3) for 30 minutes at room temperature with rotation. Controls lacking a primary antibody were subjected to the same conditions but incubated in wash buffer without any primary antibody prior to incubation with pA-MN/pA/G-MN. After incubation, samples were washed twice with was buffer and equilibrated to 0°C in an ice/water bath. MNase cleavage was activated upon addition of 3 mM CaCl_2_. Digestion was chelated for samples containing primary antibody K27ac, after 30 mins on the ice water bath, using 20 mM EDTA, 4 mM EGTA, 200 mM NaCl, and 1.5 pg MNase-digested *S. cerevisiae* mononucleosomes were added as a spike-in control. Genomic fragments were released after an RNase A treatment. Released fragments were separated through centrifugation. Digestion was chelated for samples containing primary antibodies K122ac and H3.3 after 30 mins on the ice water bath, using a low-salt treatment: 10 mM EDTA, 2 mM EGTA, 150 nM NaCl, 5 mM TritonX, and 1.5 pg MNase-digested S. cerevisiae mononucleosomes were added as a spike-in control. Genomic fragments were released after being incubated for 1 hour at 4°C and released fragments were separated from beads. The salt concentration of low salt treated samples were increased to 500 mM and additional RNase A.

Isolated fragments from all samples were used as input for a library build consisting of end repair and adenylation, adapter ligation, and subsequent purification with AMPure XP beads (Agencourt). Barcoded fragments were then amplified by 15 cycles of high-fidelity PCR and purified using agarose gel extraction. Libraries were pooled and sequenced on an Illumina NextSeq500 to a depth of ~ 10 million mapped reads.

### CUT&RUN analysis

Paired-end fastq files were trimmed to 25 bp and mapped to the mm10 genome with bowtie2^50^ (options -q -N 1 -X 1000). Mapped reads were duplicate-filtered using reads with insufficient mapping quality (MAPQ ≥ 10) were removed using samtools^43^. Reads were converted to bigWig files using deepTools^51^ with the TPM-related read normalization RPGC (options -bs 10 --normalizeUsing RPGC, --effectiveGenomeSize 2862010578). Heatmaps and metaplots were generated using deeptools. Peaks were called from CUT&RUN samples using MACS2^52^ with no antibody samples as input. For each target, peaks from all samples were combined to create a consensus peak set. After removing all peaks with less than 10 counts in 50% of samples, differential enrichment peak analysis was performed using edgeR^53^ for all available replicates of each condition with RUVseq^54^ correction. Differentially enriched peaks were defined as |log_2_(FC)|≥0.5 and FDR ≤ 0.05. For each target, samples peak annotations and motif enrichment analysis were performed using HOMER^55^.
